# Polymorphisms of Gene Cassette Promoters of the Class 1 Integron in Clinical *Proteus* Isolates

**DOI:** 10.3389/fmicb.2019.00790

**Published:** 2019-04-24

**Authors:** Linlin Xiao, Xiaotong Wang, Nana Kong, Mei Cao, Long Zhang, Quhao Wei, Weiwei Liu

**Affiliations:** ^1^Shanghai University of Medicine & Health Sciences Affiliated Sixth People’s Hospital South Campus, Shanghai, China; ^2^Department of Laboratory Medicine, Affiliated Sixth People’s Hospital South Campus, Shanghai Jiaotong University, Shanghai, China; ^3^Department of Laboratory Medicine, Shanghai Tenth People’s Hospital, Tongji University, Shanghai, China; ^4^Anhui University of Science and Technology, Anhui, China; ^5^Centre of Laboratory Medicine, Zhejiang Provincial People’s Hospital, Hangzhou, China; ^6^Department of Laboratory Medicine, Shanghai Skin Disease Hospital, Tongji University, Shanghai, China; ^7^Department of Laboratory Medicine, Shanghai First People’s Hospital, Shanghai Jiaotong University, Shanghai, China

**Keywords:** integron, gene cassettes, promoter, beta-lactamase genes, PMQR

## Abstract

**Objective:**

To describe the polymorphisms of gene cassette promoters of the class 1 integron in clinical *Proteus* isolates and their relationship with antibiotic resistance.

**Methods:**

Polymorphisms of the gene cassette promoter in 153 strains of *Proteus* were analyzed by PCR and nucleotide sequencing. Variable regions of atypical class 1 integrons were detected by inverse PCR and nucleotide sequencing. Enterobacterial repetitive intergenic consensus (ERIC)-PCR was used to analyze the phylogenetic relationships of class 1 integron-positive clinical *Proteus* isolates. Representative beta-lactamase genes (*bla*), including *bla*_TEM_,*bla*_SHV_,*bla*_CTX-M-1_,*bla*_CTX-M-2_,*bla*_CTX-M-8_,*bla*_CTX-M-9_,*bla*_CTX-M-25_ and *bla*_OXA-1_, and plasmid-mediated quinolone resistance (PMQR) genes including *qnrA, qnrB, qnrC, qnrD, qnrS, oqxA, oqxB, qepA*, and *aac(6′)-Ib* were also screened using PCR and sequence analysis.

**Results:**

Fifteen different gene cassette arrays and 20 different gene cassettes were detected in integron-positive strains. Of them, *aadB-aadA2* (37/96) was the most common gene cassette array. Two of these gene cassette arrays (*estX-psp-aadA2-cmlA1, estX-psp-aadA2-cmlA1-aadA1a-qacI-tnpA-sul3*) have not previously been reported. Three different Pc-P2 variants (PcS, PcW_TGN-10_, PcH1) were detected among the 96 *Proteus* strains, with PcH1 being the most common (49/96). Strains carrying the promoters PcS or PcW_TGN-10_ were more resistant to sulfamethoxazole, gentamicin and tobramycin than those carrying PcH1. Strains with weak promoter (PcH1) harbored significantly more intra- and extra-integron antibiotic resistance genes than isolates with strong promoter (PcW_TGN-10_). Further, among 153 isolates, representative beta-lactamase genes were detected in 70 isolates (*bla*_TEM-1_, 54; *bla*_OXA-1_, 40; *bla*_CTX-M-3_, 12; *bla*_CTX-M-14_, 12; *bla*_CTX-M-65_, 5; *bla*_CTX-M-15_, 2) and representative PMQR genes were detected in 87 isolates (*qnrA*, 6; *qnrB*, 3; *qnrC*, 5; *qnrD*, 46; *qnrS*, 5; *oqxA*, 7; *aac(6′)-Ib*, 13; *aac(6′)-Ib-cr*, 32).

**Conclusion:**

To the best of our knowledge, this study provides the first evidence for polymorphisms of the class 1 integron variable promoter in clinical *Proteus* isolates, which generally contain relatively strong promoters. Resistance genotypes showed a higher coincidence rate with the drug-resistant phenotype in strong-promoter-containing strains, resulting in an ability to confer strong resistance to antibiotics among host bacteria and a relatively limited ability to capture gene cassettes. Moreover, strains with relatively weak integron promoters can “afford” a heavier “extra-integron antibiotic resistance gene load”. Furthermore, the gene cassettes *estX*, *psp* and the gene cassette arrays *estX-psp-aadA2-cmlA1, estX-psp-aadA2-cmlA1-aadA1a-qacI-tnpA-sul3* have been confirmed for the first time in clinical *Proteus* isolates. Beta-lactamase genes and PMQR were investigated, and *bla*_TEM-1_ and *bla*_OXA-1_ were the most common, with *qnrD* and *aac (6′)-Ib-cr* also being dominant.

## Introduction

*P. mirabilis* is an important causative pathogen of various community and healthcare-associated infections, such as wound infections, primary bacteremia, pneumonia and urinary tract infections, particularly among patients with anatomical or functional urinary tract abnormalities or indwelling urinary catheters (Ahn et al., 2017). The incidence of antimicrobial resistance to *P. mirabilis* has increased, and the prevalence of *P. mirabilis* strains producing extended-spectrum β-lactamases (ESBLs), AmpC β-lactamases, carbapenemases or integrons has increased worldwide (Rzeczkowska et al., 2012). However, the impact of these elements in *P. mirabilis* infections on antimicrobial resistance is unclear. The extensive use of antibiotics leads to increased selection pressures, resulting in the emergence of antibiotic-resistant bacterial strains. Integration of exogenous antibiotic resistance genes (Guerin et al., 2009; Grieb et al., 2017) via site-specific recombination is an important pathway in the development of clinical antibiotic-resistant strains. Class 1 integrons are highly mobile and repetitive bacterial elements that integrate foreign gene cassettes and promote the expression of genes in the gene cassettes (Frumerie et al., 2010; Loot et al., 2012; Nivina et al., 2016). In addition, class 1 integrons can be integrated into chromosomes, plasmids, or transposons, carrying resistance genes with them, therefore play an important role in the formation and dissemination of drug-resistant bacterial strains (Collis et al., 2002; Ghazi et al., 2015; Makena et al., 2015; Moyo et al., 2015). The classical structure of class 1 integrons includes an integrase gene *intI1*, a recombination site *attI1*, an integrase gene transcription promoter, a *lex*A-binding site that regulates integrase gene expression, and a variable region gene cassette promoter (Collis et al., 1998, 2002; Collis and Hall, 2004; Demarre et al., 2007).

Gene cassettes in the class 1 integron usually do not include their own promoter, and their transcription depends on the common promoters Pc and P2 (Subedi et al., 2018). Several kinds of Pc variants have been defined in class 1 integrons based on their -35 and -10 hexamer sequences, and the relative strengths of these Pc variant promoters have been verified experimentally. In addition to the Pc promoter, some class 1 integrons also contain a second co-promoter P2, located about 90 bp downstream of Pc, which inserts three G residues between the -35 and -10 hexamer sequences, thus increasing the number of spaced bases to 17 bp, representing an active P2 promoter (Lévesque et al., 1994; Brizio et al., 2006; Papagiannitsis et al., 2009; Vinue et al., 2011; Moura et al., 2012). A recent study reported a new P2 promoter variant, P2m3, with a similar strength to the PcW_TGN-10_ variant (Lin et al., 2017). Jove et al. (2010) described variants of various types of Pc promoters, and noted that promoter polymorphisms could result in changes in the amino acid species in the IntI1 sequence, with the magnitude of the change in the excision activity of the mutant integrase being greater than the magnitude of the change in its integration activity. In addition, given identical Pc promoters, the integration efficiency is significantly reduced if the P2 promoter is located before the *attI1* site (Guerin et al., 2011). Guerin et al. (2011) carried out a detailed study of the transcriptional interference relationship between the *intI1* promoter PintI1 and the Pc or Pc-P2 combination and showed that higher gene cassette transcription levels inhibited expression of the integrase in class 1 integrons. The Pc and P2 co-promoter of class 1 integrons therefore not only play an important role in driving the transcription of downstream gene cassettes or gene cassette arrays, but also have a close relationship with the resection and integration phenomena that occur during the capture of exogenous gene cassettes. However, no promoter-related studies of class 1 integrons in clinical isolates of *Proteus* have yet been reported. In this study, we investigated the polymorphisms of the co-promoter of class 1 integrons and their association with the antibiotic resistance phenotype in clinical isolates of *Proteus*.

## Materials and Methods

### Bacterial Strains and Susceptibility Testing

We previously obtained 153 strains of *Proteus* from patient samples from Zhejiang Province (Wei et al., 2014). These clinical isolates included 140 *P. mirabilis* isolates, 12 *Proteus vulgaris* isolates and 1 *Proteus penneri* isolate. Among these, 96 class 1 integron positive strains were studied further. *Escherichia coli* ATCC25922 and *E. coli* DH5α were also maintained in our laboratory. Antibiotic susceptibility was determined by disk diffusion and broth dilution. *E. coli* ATCC25922 was used as a control strain. The tested antibiotics included: amikacin, gentamicin, tobramycin, sulfamethoxazole, chloramphenicol, Meropenem, Imipenem, Ciprofloxacin, Levofloxacin, Aztreonam, Cefepime, Ceftriaxone, Ceftazidime, Cefotetan, and Cefazolin. The results were interpreted in accordance with the guidelines of the Clinical and Laboratory Standards Institute.

### Structural Analysis of Atypical Class 1 Integrons

**FIGURE 1:**
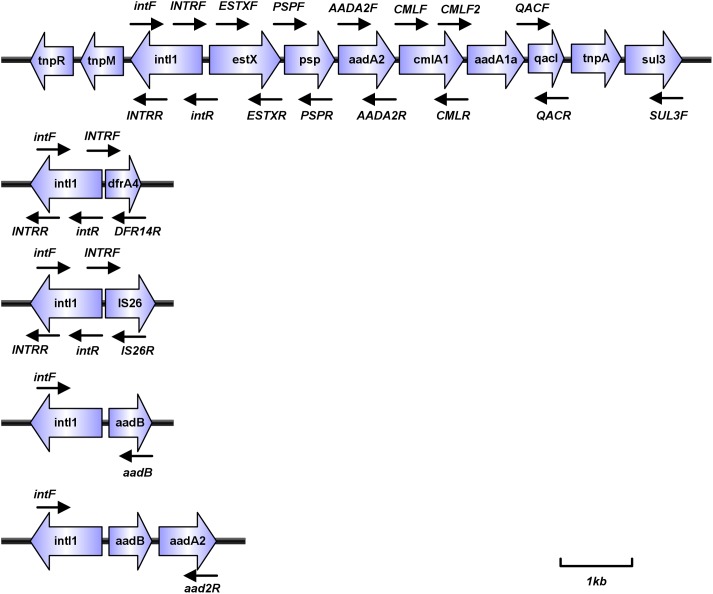
PCR scheme (thin black arrows indicate the position of primer; thick blue arrows represent different genes).

Bacterial DNA preparation and class 1 integron analysis were conducted and reported as previously (Wei et al., 2014). Variable regions of atypical class 1 integrons that could not be amplified conventionally were detected by inverse PCR analysis of genomic DNA using the primer pairs INTRR and INTRF, followed by verification by electrophoresis and sequencing (Table 1 and Figure 1). For *aac(6′)-Ib* gene positive isolates, the variable regions were also amplified through overlap PCR using the primer pairs intF and aacR, aacF and 3CS. PCR products were analyzed by sequencing. All sequencing results were aligned using the BLAST program^1^.

**Table 1:** Primers used for PCR amplification.

Primer	Primer sequence(5′–3′)	References
intF	CCAAGCTCTCGGGTAACATC	Wei et al., 2011
P2R2	CCCGAGGCATAGACTGTA	Sunde, 2005
ERIC2	AAGTAAGTGACTGGGGTGAGCG	Tsai et al., 2018
3CS	AAGCAGACTTGACCTGA	Sunde, 2005
INTRF	TCGGCCATTCCGACGTCTCTAC	Sunde, 2005
INTRR	TGCAAGTAGCGTATGCGCTC	Sunde, 2005
CMLF	AAACGCGCTTGGTACGACAGC	This study
CMLR	ATTACTTTCCTCGCGACCTGC	This study
AADA2F	CGATGAGCGAAATGTAGTG	This study
AADA2R	AAGACGGGCTGATACTGG	This study
ESTXF	AGGTCAGGCTCCATATTCC	This study
ESTXR	TGAATGTTGTCAGGATATTC	This study
QACF	TTGGTGAGGTCGTCGCAAC	This study
QACR	TGCGCTGACCTTGGATAGC	This study
SUL3F	GAGCAAGATTTTTGGAATCG	This study
PSPF	TCGATGGCACAATTACCAC	This study
QD14R1	CCTGAGCGGGTAACGAC	This study
IS26R	TTGCGTAGTGCACGCATCACC	This study
CMLF2	TAGGTTTGGGCATGATC	This study
TEMF	TCGGGGAAATGTGCG	Velasova et al., 2019
TEMR	TGCTTAATCAGTGAGGCACC	Velasova et al., 2019
SHVF	GCCTTTATCGGCCTTCACTCAAG	Velasova et al., 2019
SHVR	TTAGCGTTGCCAGTGCTCGATCA	Velasova et al., 2019
CTX-M-1F	CAGAGATTTTGCCGTCTAAG	Velasova et al., 2019
CTX-M-1R	GGCCCATGGTTAAAAAATCACTGC	Velasova et al., 2019
CTX-M-2F	CTCAGAGCATTCGCCGCTCA	Velasova et al., 2019
CTX-M-2R	CCGCCGCAGCCAGAATATCC	Velasova et al., 2019
CTX-M-8F	ACTTCAGCCACACGGATTCA	Velasova et al., 2019
CTX-M-8R	CGAGTACGTCACGACGACTT	Velasova et al., 2019
CTX-M-9F	GTTACAGCCCTTCGGCGATGATTC	Velasova et al., 2019
CTX-M-9R	GCGCATGGTGACAAAGAGAGTGCAA	Velasova et al., 2019
CTX-M-25F	GCACGATGACATTCGGG	Velasova et al., 2019
CTX-M-25R	AACCCACGATGTGGGTAGC	Velasova et al., 2019
OXA-1-F	GGCACCAGATTCAACTTTCAAG	Che et al., 2014
OXA-1-R	GACCCCAAGTTTCCTGTAAGTG	Che et al., 2014
qnrAF	AGAGGATTTCTCACGCCAGG	Kim et al., 2016
qnrAR	GCAGCACTATKACTCCCAAGG	Kim et al., 2016
qnrBF	GGMATHGAAATTCGCCACTG	Kim et al., 2016
qnrBR	TTTGCYGYYCGCCAGTCGAA	Kim et al., 2016
qnrCF	GGGTTGTACATTTATTGAATC	Kim et al., 2016
qnrCR	TCCACTTTACGAGGTTCT	Kim et al., 2016
qnrDF	CGAGATCAATTTACGGGGAATA	Kim et al., 2016
qnrDR	AACAAGCTGAAGCGCCTG	Kim et al., 2016
qnrSF	GCAAGTTCATTGAACAGGCT	Kim et al., 2016
qnrSR	TCTAAACCGTCGAGTTCGGCG	Kim et al., 2016
oqxAF	GACAGCGTCGCACAGAATG	Wong et al., 2014
oqxAR	GGAGACGAGGTTGGTATGGA	Wong et al., 2014
oqxBF	CGAAGAAAGACCTCCCTACCC	Kim et al., 2016
oqxBR	CGCCGCCAATGAGATACA	Kim et al., 2016
qepAF	CTGCAGGTACTGCGTCATG	Wong et al., 2014
qepAR	CGTGTTGCTGGAGTTCTTC	Wong et al., 2014
aacF	ATCTCATATCGTCGAGTGG	This study
aacR	TGCGTGTTCGCTCGAATGC	This study


### Characterization of Pc and P2 Promoters of Class 1 Integrons

For typical class 1 integrons, the type of promoter upstream of the variable region was identified by direct sequencing. For atypical class 1 integrons, Pc and P2 promoters were identified by sequencing the PCR products amplified using the primer intF combined with specific primers for the downstream gene cassettes. For strains that cannot be successfully amplified using intF and specific primers for the downstream gene cassette, the class 1 integron-mixed common promoter was amplified only by intF and P2R2 primer pairs (some strains may contain multiple integrons). All of them were sequenced using the primer intF after electrophoresis validation, and the variable region promoter type was interpreted based on the sequence.

### Polymerase Chain Reaction Detection and Sequencing of Beta-Lactamase Genes

To determine the genotype of beta-lactamase, we performed PCR amplification with *bla*_TEM_,*bla*_SHV_,*bla*_CTX-M-1_,*bla*_CTX-M-2_, *bla*_CTX-M-8_,*bla*_CTX-M-9_,*bla*_CTX-M-25_, and *bla*_OXA-1_. Specific primers that were designed to detect beta-lactamase gene markers (Table 1) were used to screen for beta-lactamase antibiotic resistance gene in bacterial isolate template DNA. The total volume of the PCR mixture was 20 μl, containing 1 μl of genomic DNA template, 0.4 μl of each primer (10 pmol), 10 μl of Premix-rTaq PCR solution (TaKaRa, Japan), and 7 μl of distilled water. PCR was carried out using an ABI Veriti Thermal Cycler (Applied Biosystems, Singapore). The template was initially denatured at 94°C for 4 min, followed by 35 cycles of 94°C for 40 s, 55°C for 40 s, and 72°C for 40 s, with a final extension at 72°C for 5 min. PCR products were verified by electrophoresis and sequencing (Table 1). All sequencing results were aligned using the BLAST program.

### Multiplex PCR Detection of Plasmid-Mediated Quinolone Resistance Genes

To determine the genotype of plasmid-mediated fluoroquinolone resistance genes, we performed PCR amplification with the *qnrA* (length = 619 bp), *qnrB* (length = 264 bp), *qnrC* (length = 447 bp), *qnrD* (length = 582 bp), *qnrS* (length = 428 bp), *oqxA* (length = 339bp), *oqxB* (length = 240 bp), *qepA* (length = 403 bp), and *aac(6′)-Ib*, *qnrA*/*qnrB*/*qnrC* as the first multiplex PCR amplification system, and *qnrD*/*qnrS*/*oqxA*/*oqxB* as the second multiplex PCR amplification system. *qepA* and *aac(6′)-Ib* were separately amplified. Specific primers which were designed for fluoroquinolone resistance maker (Table 1) were used to screen for antibiotic resistance genes in bacterial isolate template DNA. PCR amplification components and cycling conditions were identical to those used for the detection of BLA antibiotic resistance genes described above, followed by verification by electrophoresis. All *aac(6′)-Ib* positive products were then sequenced (Table 1). All sequencing results were aligned using the Vector NTI Advance 11 (Invitrogen, United States).

### Determination of Phylogenetic Groups of *Proteus*

We analyzed the phylogenetic population of the 96 integron-positive *Proteus* strains based on the Enterobacterial repetitive intergenic consensus (ERIC)-PCR method (Wilson and Sharp, 2006). Phylogenetic groups of *Proteus* strains were determined according to the electrophoresis patterns of the PCR product by NTSYSpc 2.1e software (clustering program).

### Statistical Analysis

All statistical analyses were performed using SPSS software, version 22.0. To compare the two groups, the Student’s *t*-test or Mann-Whitney *u*-test, depending on the validity of the normality assumption, was used for continuous variables. The chi-squared test or Fisher’s exact test was used to assess categorical variables. Values of *p* < 0.01 were considered to indicate significance.

## Results

### Antimicrobial Susceptibility

In this study, 153 strains of *Proteus* were isolated mainly from patients in the Internal Medicine surgery ward [53.6% (82/153)], the ICU [37.3% (57/153)] and the Outpatient clinic [9.1% (14/153)]. The cohort of 153 patients had a mean age of 67.2 years, which a range of 5–91. 104 (68.0%) patients were over 60 years old. The main sources of *Proteus* were from genital secretions [17.6% (27/153)], urine [41.2% (63/153)], sputa [32.7% (50/153)], hydrothorax and ascite [5.9% (9/153)], and blood [2.6% (4/153)].

The *in vitro* antimicrobial susceptibilities of the *Proteus* isolates showed that most isolates were susceptible to Imipenem (60%), Meropenem (55.6%), Ciprofloxacin (40.5%), Levofloxacin (52.3%), Cefepime (63.4%), Ceftriaxone (58.8%), Ceftazidime (58.2%), Cefazolin (41.8%), Aztreonam (79.1%), Amikacin (81.7%), Gentamicin (47.1%), Tobramycin (45.6%), Sulfamethoxazole (43.1%), and Chloramphenicol (61.4%). Moreover, all of the isolates were susceptible to Piperacillin/Tazobactam and Cefotetan.

### Characterization of Gene Cassettes and Arrays

**FIGURE 2:**
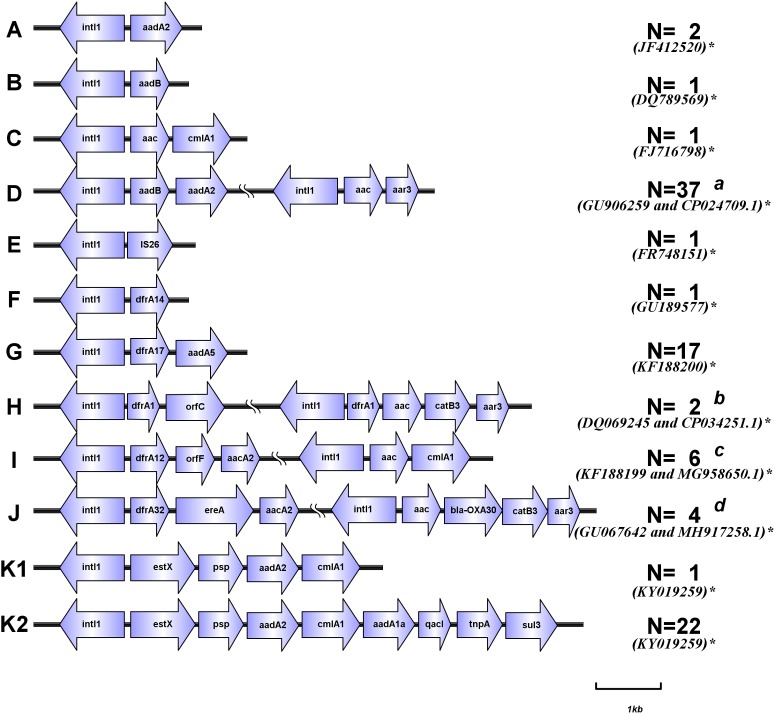
Schematic representation of different types of gene cassette arrays (*aac* equal to *aac(6′)-Ib* or *aac(6′)-Ib-cr*). ^∗^Accession numbers in Genbank. ^a^Among the 37 isolates, 25 of the integron variable regions were *aadB-aadA2*, and the other 12 were *aadB-aadA2* and *aac(6′)-Ib-aar3*. ^b^Among the 2 isolates, 1 of the integron variable regions were *dfrA1-orfC*, and the other 1 was *dfrA1-orfC* and *dfrA1- aac(6′)-Ib -catB3-aar3.* ^c^Among the 6 isolates, 5 of the integron variable regions were *dfrA12-orfF-aacA2*, and the other 1 was *dfrA12-orfF-aacA2* and *aac(6′)-Ib -cmlA1*, the second array (*aac(6′)-Ib -cmlA1*) is the same as C(*aac(6′)-Ib -cmlA1*). ^d^Among the 4 isolates, 3 of the integron variable regions were *dfrA32-ereA-aacA2*, and the other 1 was *dfrA32-ereA-aacA2* and *aac(6′)-Ib -bla-_OXA-1_-catB3-aar3*. (Sequences of PCR products were analyzed with BLAST to identify target homologous sequences and their GenBank accession numbers. https://blast.ncbi.nlm.nih.gov/Blast.cgi).

Of 96 class 1 integrin-positive strains, 70 variable regions of typical integrons were previously detected in *Proteus* strains (Wei et al., 2013). Variable regions in 26 atypical class 1 integrons were analyzed using inverse PCR. For *aac(6′)-Ib* gene positive isolates, the variable regions were amplified through overlap PCR. A total of 15 different types of variable region gene cassette arrays and 20 different gene cassettes were detected. These gene cassette arrays were divided into types A–K, of which type K included K1 and K2 (Figure 2). The most common antibiotic resistance gene cassettes were *aadA2* (72/96), *aadB* (38/96), and *aadA1a* (22/96), all of which conferred resistance to aminoglycoside antibiotics. Five trimethoprim-resistance gene cassettes [*dfrA17* (17/96), *dfrA12* (6/96), *dfrA32* (4/96), *dfrA1* (2/96), *dfrA14* (1/96)] conferred resistance to trimethoprim antibiotics; in addition, we also found *aac(6′)-Ib* gene cassettes (16/96) in the integron variable region, and a chloramphenicol-resistance gene cassette *cmlA1* (2/96). The gene cassette arrays were partly detected in strain NO.47685 (*IS26*) and strain NO.50772 (*dfrA14*), but variable regions were not detected in strain NO.45016 (Table 2). The most common gene cassette arrays were *aadB-aadA2*, *estX-psp-aadA2-cmlA1-aadA1a-qacI-tnpA-sul3*, and *dfrA17-aadA5*, which were detected in 37, 22, and 17 isolates, respectively.

**Table 2:** Gene cassette arrays and their common promoters in 96(class 1 integrons)*Proteus* strains.

Gene cassette array	Type promoter			Total
		
	PcH1	PcW_TGN-10_	PcS	
*aadA2*^∗^			2	2
*aadB*^∗^		1		1
*aac(6′)-Ib -cmlA1*^∗^		1		1
*aadB-aadA2*^∗^			37	37
*IS26^a^*	1			1
*dfrA14^a^*	1			1
*dfrA17-aadA5*^∗^	17			17
*dfrA1-orfC*^∗^		2		2
*dfrA12-orfF-aadA2*^∗^	2	3	1	6
*dfrA32-ereA-aadA2*^∗^	4			4
*estX-psp-aadA2-cmlA1*	1			1
*estX-psp-aadA2-cmlA1-aadA1a-qacI-tnpA-sul3*	22			22
Unknown*^b^*	1			1
*aac(6′)-Ib -aar3*			12	12
*aac(6′)-Ib -bla*_OXA-1_*-catB3-aar3*	1			1
*dfrA1- aac(6′)-Ib -catB3-aar3*		1		1
Total	50	8	52	110*^c^*


*^*a*^Class 1 integrons for which only partial sequences of gene cassette arrays were amplified (IS26 detected in NO.47685 strain, dfrA14 detected in NO.45016 strain). ^*b*^Class 1 integrons for which PCR failed to amplify the gene cassette array. ^*c*^A total of 110 integrants were detected from 96 integron positive strains. ^∗^The gene cassette combination that our research group has previously reported.*

### Class 1 Integron Promoter Variants

We analyzed the promoters of class 1 integrons. All bacterial strains are shown in Table 2. Three common types of promoters were detected among the 96 clinical isolates of integron-positive *Proteus* strains. The most common promoter was PcH1, which was a relatively weak promoter occurring in 51% (49/96) of class 1 integron-positive strains (Wei et al., 2011), while PcS was the second most prevalent promoter, present in 41.6% (40/96), and the PcW_TGN-10_ was detected in only 7.3% (7/96) of class 1 integron-positive strains. An inactive P2 promoter unable to drive the expression of downstream gene cassettes was detected in all class 1 integron-positive strains.

Regarding the relationship between gene cassettes or gene cassette arrays and specific common promoters, PcH1 could drive the expression of *estX-psp-aadA2-cmlA1-aadA1a-qacI-tnpA-sul3*, *dfrA17-aadA5*, *dfrA32-ereA-aadA2*, and *estX-psp-aadA2-cmlA1* gene cassette arrays, PcS could drive *aadB-aadA2*, *aadB*, and *aadA2* gene cassette arrays, and PcW_TGN-10_ could drive the expression of *dfrA1-orfC* and *aacA4-cmlA1* gene cassette arrays. In addition, all three types of promoters (PcS, PcH1, and PcW_TGN-10_) could drive the expression of the gene cassette array *dfrA12-orfF-aadA2*.

### Associations Between Common Promoter Variants and Phylogenetic Groups of *Proteus*

**FIGURE 3:**
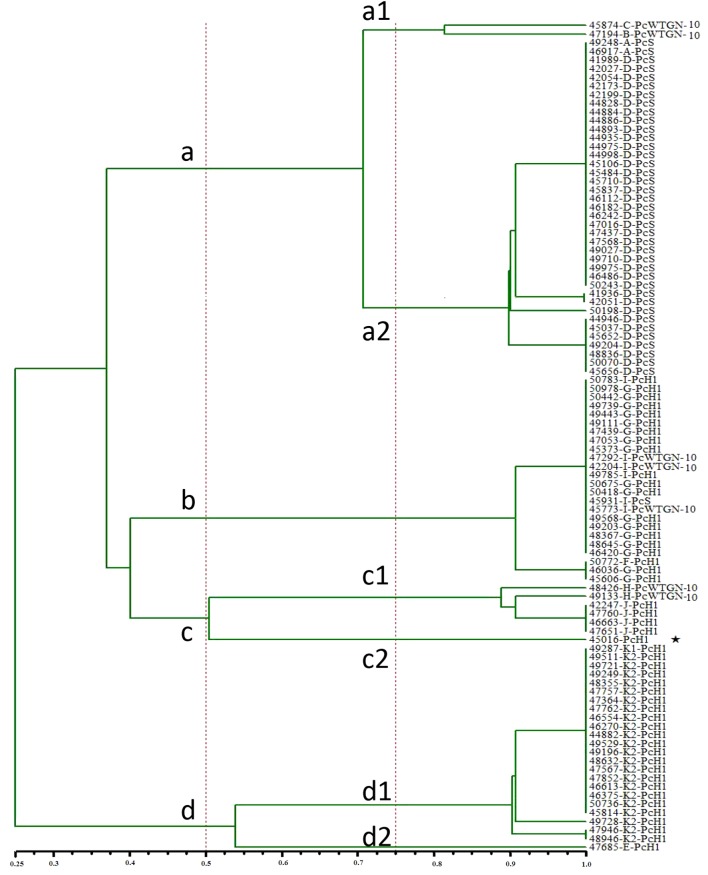
Cluster analysis of 96 strains of Proteus based on ERIC-PCR. (50% homology gathers as a main class, and 75% homology gathers into a subclass, 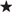
Class 1 integrons for which PCR failed to amplify the gene cassette array).

We analyzed the phylogenetic relationships between clinical isolates of *Proteus*. We divided the 96 clinical isolates of class 1 integron-positive *Proteus* into seven groups (a1, a2, b, c1, c2, d1, and d2) according to the ERIC-PCR results. Among these, two strains belonged to group a1 [PcW_TGN-10_ (2/2)], 39 to group a2 [PcS (39/39)], 24 to group b [PcH1 (20/24), PcW_TGN-10_ (3/24), PcS (1/24)], six to group c1 [PcH1 (4/6), PcW_TGN-10_ (2/6)], one to group c2 (PcH1), 23 to group d1 [PcH1 (23/23)], and one to group d2 (PcH1) (Figure 3). The a1, a2, and d1 groups each included a single promoter type. The c2 (strain NO.45016) and d2 groups (strain NO.47685) each included only one strain, among which the integron variable region of 45016 could not be detected and the integron variable region of 47685 was an insertion sequence (*IS26*), which was different from that of other strains.

### Relationships Between *Proteus* Pc and Pc-P2 Promoters and Resistance Phenotype

**FIGURE 4:**
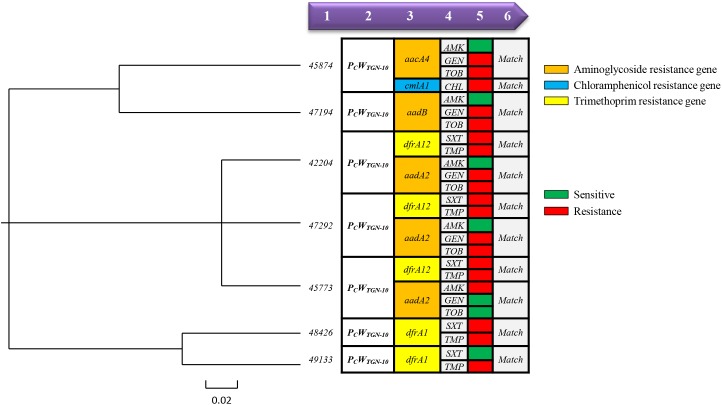
Relationships between resistance gene and resistance phenotype in *Proteus* strains with strong promoters. (On the left side of this image is a phylogenetic tree based on the DNA sequence of each strain promoter using Vector NTI Advance 11 software, while the right side of this image shows the relationship between the variable region genotype and the resistant phenotype of each strain.) ^1^Strain number. ^2^Promoter type. ^3^Integron variable region genotype (aminoglycoside resistance gene shown in orange, chloramphenicol resistance gene shown in blue and trimethoprim resistance gene shown in yellow). ^4and5^Phenotype (sensitive shown in green, resistance shown in red). ^6^Indicates whether the genotype matches the resistant phenotype (at least one genotype corresponds to a resistant phenotype and is considered to be a “*Match*”).

We tested the 96 class 1 integron-positive *Proteus* strains for antibiotic susceptibility, to clarify the relationship between the integron variable region promoter and the antibiotic-resistance phenotype in clinical isolates. Integron-positive strains containing relatively strong promoters had higher resistance rates to amikacin, gentamicin, and tobramycin, but low resistance to chloramphenicol (Table 3). There was no significant difference in sulfamethoxazole and chloramphenicol resistance rates between strains with relatively strong and weak promoters. However, strains with strong promoters still had higher MIC_50_ values for chloramphenicol than strains with weak promoters. We performed a more detailed analysis of the promoters and antibiotic-resistance phenotypes in the seven strains of bacteria with strong promoters (PcW_TGN-10_) and showed that resistance phenotype was associated with the presence of a strong promoter (PcW_TGN-10_), while this phenomenon was not observed in other promoter types (Figure 4).

**Table 3:** Associations of promoter variants with antibiotic-resistance phenotypes.

Promoter	Total no. of isolates	No. (%) of isolates with resistance to*^a^*				
		
		AMK	GEN	TOB	SXT	CHL
Total	96	12 (12.5)	51 (53.1)	39 (40.6)	69 (71.9)	39 (40.6)
Strong promoter	47	12 (25.5)	38 (82.8)	37 (78.7)	37 (78.7)	17 (36.2)
PcW_*TGN*-10_	7	1 (14.3)	5 (71.4)	5 (71.4)	6 (85.7)	1 (14.3)
PcS	40	11 (27.5)	33 (85.2)	32 (80)	31 (77.5)	16 (40)
MIC_50_ (μg/ml)		≤ 2	≥ 16	≥16	≥ 16	≥64
Weak promoter	49	0 (0)	13 (26.5)	2 (4.1)	32 (65.3)	22 (44.9)
PcH1	49	0 (0)	13 (26.5)	2 (4.1)	32 (65.3)	22 (44.9)
MIC_50_ (μg/ml)		–	≤ 4	≤4	≥ 16	16
*p-*value*^b^*		0.000	0.000	0.000	0.144	0.384


*^a^AMK amikacin, GEN gentamicin, TOB tobramycin, SXT Trimethoprim/Sulfamethoxazole, CHL chloramphenicol ^*b*^The chi-squared test was used to assess strong promoter and weak promoters.*

### Genotypes of Beta-Lactamase Genes

Among the beta-lactamase producing strains, we found 55 isolates that were positive for *bla*_TEM_, 15 isolates positive for the *bla*_CTX-M-1_ group, 17 isolates positive for the *bla*_CTX-M-9_ group and 40 isolates positive for the *bla*_OXA-1_ group. Using nucleotide sequence analysis, we found that 55 *bla*_TEM_ positive isolates carried *bla*_TEM-1_. Of 15 *bla*_CTX-M-1_ group positive isolates, 12 had *bla*_CTX-M-3_ and 3 carried *bla*_CTX-M-15_. Meanwhile, of 17 *bla*_CTX-M-9_ group positive isolates, 12 had *bla*_CTX-M-14_ and 5 had *bla*_CTX-M-65_. All 40 *bla*_OXA-1_ group positive isolates were found to carry *bla*_OXA-1_. Meanwhile, all 153 isolates were negative for *bla*_SHV_, *bla*_CTX-M-2_ group, *bla*_CTX-M-8_ group and *bla*_CTX-M-25_ group. Statistical analysis of the drug-sensitive phenotypes of the beta-lactamase positive and negative-positive groups revealed that the beta-lactamase positive group was significantly less sensitive to Ceftriaxone (35.7% vs. 77.1%, *p* < 0.01), Ceftazidime (31.4% vs. 80.7%, *p* < 0.01), Cefazolin (38.6% vs. 84.3%, *p* < 0.01), Imipenem (37.1% vs. 79.51%, *p* < 0.01), and Meropenem (35.71% vs. 72.3%, *p* < 0.01) than the beta-lactamase negative group (Table 4).

**Table 4:** *In vitro* antimicrobial susceptibility of *bla* and PMQR.

Antimicrobial agent	No. (%) of susceptible			No. (%) of susceptible		
		
	*bla* positive	*bla* negative	*p-*value	PMQR positive	PMQR negative	*p-*value
	***N* = 70**	***N* = 83**		***N* = 87**	***N* = 66**	
Imipenem	26 (37.1)	66 (79.5)	0.000	52 (59.8)	40 (60.6)	0.917
Meropenem	25 (35.7)	60 (72.3)	0.000	55 (63.2)	40 (60.6)	0.742
Ciprofloxacin	22 (31.4)	40 (48.2)	0.035	19 (21.8)	43 (65.2)	0.000
Levofloxacin	33 (47.1)	47 (56.6)	0.242	29 (33.3)	51 (77.3)	0.000
Cefepime	27 (38.6)	70 (84.3)	0.000	57 (65.5)	40 (60.6)	0.532
Ceftriaxone	25 (35.7)	64 (77.1)	0.000	57 (65.5)	32 (48.5)	0.034
Ceftazidime	22 (31.4)	67 (80.7)	0.000	60 (68.9)	29 (43.9)	0.002
Cefazolin	20 (28.5)	44 (53.0)	0.002	36 (41.4)	28 (42.4)	0.897
Aztreonam	51 (72.8)	70 (84.3)	0.082	73 (83.9)	48 (72.7)	0.092
Amikacin	54 (77.1)	71 (85.5)	0.181	78 (89.7)	47 (71.2)	0.003
Gentamicin	28 (40.0)	44 (53.0)	0.108	39 (44.8)	33 (50.0)	0.526
Tobramycin	28 (40.0)	42 (50.6)	0.190	39 (44.8)	31 (47.0)	0.792
Sulfamethoxazole	18 (25.7)	48 (57.8)	0.000	30 (34.5)	36 (54.5)	0.013
Chloramphenicol	41 (58.6)	53 (63.9)	0.504	47 (54.0)	47 (71.2)	0.031
Piperacillin/Tazobactam	70 (100)	83 (100)	–	87 (100)	66 (100)	–
Cefotetan	70 (100)	83 (100)	–	87 (100)	66 (100)	–


### Plasmid-Mediated Quinolone Resistance Gene

Among 153 *Proteus* samples, we found 6 isolates positive for *qnrA*, 3 isolates positive for *qnrB*, 5 isolates positive for *qnrC*, 46 isolates positive for *qnrD*, 5 isolates positive for *qnrS*, 7 isolates positive for *oqxA* and 45 isolates positive for *aac (6′)-Ib*, while all 153 isolates were negative for *oqxB* and *qepA*. All *aac (6′)-Ib* positive products were detected using nucleotide sequence analysis, and we found two types of the *aac (6′)-Ib* gene, which were *aac (6′)-Ib* (13/45) *and aac (6′)-Ib-cr* (32/45). Statistical analysis of the drug-sensitive phenotypes of the PMQR positive and negative groups showed that the PMQR positive group was significantly less sensitive to Ciprofloxacin (21.8% vs. 65.2%, *p* < 0.01) and Levofloxacin (33.3% vs. 77.3%, *p* < 0.01) than the PMQR negative group (Table 4).

### Relationships Between Various Promoters and Antibiotic Resistance Gene Load

We compared the antibiotic resistance gene load of different promoters of 96 integron positive strains. We found that the relatively weak promoter (PcH1) strains carried 6.88 resistance genes on average, of which 5.35 resistance genes were located in the integrons, and there were 1.53 resistance genes not located on the integrons (including: 1.12 beta-lactamase genes, 0.41 PMQR). The relatively strong promoter (PcW_TGN-10_ and PcS) strains carried 3.57 and 3.88 resistance genes on average, respectively. Simultaneously, on average, 2.57 and 2.55 antibiotic resistant genes were located on integrons, while 1 (including: 0.85 beta-lactamase genes, 0.15 PMQR) and 1.3 (including: 0.9 beta-lactamase genes, 0.4 PMQR) antibiotic-resistant genes were not located on integrons, respectively (Table 5).

**Table 5:** Associations of promoter variants with gene load.

Promoter		Total no. of isolates	No. of antibiotic resistance genes				
			
			No. of genes	Located on integrons	Not located on integrons	*bla*	PMQR
“Strong” promoter	PcW_TGN-10_	7	3.57*^a^*	2.57*^a^*	1*^a^*	0.85*^a^*	0.15*^a^*
	PcS	40	3.85*^a^*	2.55*^a^*	1.3*^a^*	0.9*^a^*	0.4*^a^*
“Weak” promoter	PcH1	49	6.88*^a^*	5.35*^a^*	1.53*^a^*	1.12*^a^*	0.41*^a^*


*^*a*^Average value.*

## Discussion

Integrons are genetic elements with a specific functional configuration that have evolved in bacteria and which can capture and express exogenous gene cassettes via site-specific recombination. In this study, 96 strains containing class 1 integrons were detected among 153 clinical isolates of *Proteus*, indicating that this evolutionary platform is common among clinical strains. Additionally, we detected 20 different gene cassettes, most of which conferred resistance to antibiotics. Antibiotics such as trimethoprim, chloramphenicol, and erythromycin were discovered in the early and mid-20th century and are now used extensively in clinical applications. However, during the process of bacterial evolution, antibiotic resistance gene cassettes have spread throughout clinical strains due to integration subsystems and high selection pressure imposed by the combined action of a large number of antibiotics, allowing the survival of bacteria carrying the appropriate antibiotic-resistance genes.

In contrast to previous research on Pc promoter polymorphisms in *E. coli* (Wei et al., 2013), the three promoters identified in the current study were relatively strong promoters (PcS, PcW_TGN-10_, and PcH1), with the stronger promoters (PcS, PcW_TGN-10_) accounting for 49% of all integron-positive strains. The variety of integron variable region gene cassettes was also shown to be more complicated, with *estX* and *psp* being detected for the first time in clinical isolates of *Proteus*. Integrons usually spread between strains with the help of plasmids or transposons. Additionally, we detected the same array of gene cassettes in different phylogenetic groups of clinical isolates of *Proteus*, and the upstream promoters also remained stable. This may be due to the class 1 integrons being embedded in larger transposons or plasmids, or may be recombined in a conserved region of the class 1 integron 5CS, such that the gene cassette array is combined with the same promoter.

This article reveals that strains with strong promoters have higher rates of antibiotic resistance than strains with weaker promoters, especially in amikacin, gentamicin, and tobramycin. This may be explained by the presence of a strong promoter in the variable region of the class 1 integron causing high expression of the relevant antibiotic-resistant genes. Interestingly, the antibiotic-resistant genotypes and phenotypes were highly matched among the seven strains with the strong promoter PcW_TGN-10_, while strains containing other types of promoters do not show this phenomenon. In the phylogenetic analysis (Figure 3), we found that these 7 strains clearly belong to different colony groups. In summary, antibiotic genes are located close to the promoter, making it relatively easy for the promoter to regulate their expression. However, the current results were only relevant to the individual strains studied, and clinical strains with different genetic backgrounds may present more complex phenomena.

In this article, we elucidated the relationship between beta-lactamase genes and integrons that were carried in strains. Therefore, we screened the beta-lactamase resistance gene of 153 *Proteus* isolates, and found that the positive rate reached 45.8%, which was significantly higher than previous reports (Ahn et al., 2017). A crucial argument shown by the statistical results is that there is a significant difference (*p* < 0.01) in the difference in drug resistance gene carrying between beta-lactamase genes and integrons in *Proteus* strains (Table 6). As a result, we studied their impact on antibiotic resistance and attempted to explain the association between the antibiotic resistance genes carried by these strains and the integron promoter. Moreover, we found that beta-lactamase genes were significantly more detectable in ICUs and surgical wards than in other wards, as most ICU patients had severe disease, reduced immunity, and long-term use of antibiotics, all of which helped improve detection rate. For patients undergoing urologic surgery, the higher detection rate is related to its own physiological structural characteristics, one of which is mainly urinary tract obstruction, which is conducive to bacterial reproduction, in addition to urinary catheterization, further increasing the chance of infection. Furthermore, we found that most beta-lactamase producing strains occur in the elderly or women. Among the strains studied, we did not find other significant differences in gene carriers. This may be due to the low immunity of the elderly and the vulnerability of the female urethra to infection, so that some strains or resistance genes can be transmitted horizontally.

**Table 6:** The relationship between carriage of integron and *bla*, PMQR.

Genotypes	No.(%) of carried		*p-*value
		
	Integron positive	Integron negative	
	***N* = 96**	***N* = 57**	
*bla*	57	13	0.000
PMQR	48	39	0.026


TEM is the main type of β-lactamases, and the TEM-1 group is the most common. The CTX-M enzyme is a new group of plasmid-mediated beta-lactamase genes that have dominated in Europe, and have increased dramatically in many countries over the past decade (Mohd et al., 2019). Antibiotic consumption and different risk factors may also contribute to the current epidemiology of CTX-M enzymes in different geographic regions. In recent years, China has also presented an increasing trend, and there are few reports of beta-lactamase genes in *Proteus* isolated from Chinese hospitals. Interestingly, our research found that *bla*_TEM-1_, *bla*_OXA-1_, and *bla*_CTX-M-14_ were carried in the same strains, and they are resistant to third-generation cephalosporin, which may be synergy between them, increasing the ability of bacteria to hydrolyze cephalosporin. Drug susceptibility test data showed that *Proteus* producing beta-lactamase genes was significantly less sensitive to most third-generation cephalosporins (Table 4). If the patient is infected by a beta-lactamase producer, Cefotetan, Cefmetazole or Imipenem may be preferred prior to the results of the antibiotic susceptibility test, but if the patient is in a critical state, we should choose carbapenem antibiotics. These findings lead us to conclude that we should pay attention to the use of antibiotics in outpatient, inpatient and community hospitals, and reduce the chance of dissemination of β-lactamase gene levels due to antibiotic selection pressure.

The PMQR genes discovered in recent years, such as *qnrA, qnrB, qnrC, qnrD, qnrS, aac (6′)-Ib-cr*, and *qepA* resistance genes, are an important mechanism for bacteria to resist quinolone. In this study, we explored the relationship between quinolone resistance genes and integrons in *Proteus*, and we also screened quinolone resistance genes in 153 *Proteus* isolates, with a positive rate of up to 56.9%, mainly carrying *qnrD* and *aac(6′)-Ib*. Notably, *aac(6′)-Ib* is not resistant to quinolones, only variant *aac(6′)-Ib-cr* is resistant to quinolone. Among them, we studied *aac(6′)-Ib* in depth. The nucleic acid sequence of *aac(6′)-Ib* was found to contain Asp181Tyr (G541T) and Trp104Arg (T310C or T310A) in 32 strains of *aac(6′)-Ib* (Hidalgo-Grass and Strahilevitz, 2010). The variant *aac(6′)-Ib-cr* can confer bacterial resistance to Ciprofloxacin or Levofloxacin. In general, *aac(6′)-Ib* is mainly located in integrons and spreads horizontally with the spread of integrons. In this study, only 16 strains of *aac(6′)-Ib* were located in integrons (*aac(6′)-Ib-aar3*,12; *aac(6′)-Ib-bla_OXA-1_-catB3-aar3*,1; *aac(6′)-Ib-cmlA1*,1; *dfrA1-aac(6′)-Ib-catB3-aar3*,1; *aac(6′)-Ib -cmlA1*,1), and all *aac(6′)-Ib-cr* variants were located on the integrons. However, *aac(6′)-Ib*, which cannot confer PMQR, was carried by another 29 strains and may be located on other mobile elements, such as transposons or insertion sequences, although its specific mechanism of action needs further study. In the end, the experimental results were contrary to our hypothesis. There was no statistically significant difference in the quinolone resistance gene and integron carrying in the *Proteus* strains (*p* > 0.01) (Table 6).

In this study, multiple resistance genes were detected in isolates, and we also compared the antibiotic resistance “gene load” of strains with different promoters. As such, it further explains the fitness of the clinical bacteria. These results demonstrate that strains with relatively weak integron promoters can “afford” a heavier intra- and extra-integron antibiotic resistance gene load. Although many antibiotic resistance genes are not in the integrons, such as *bla* and PMQR, and are not directly related to the integron promoter, only a few representative *bla* and PMQR genes were investigated in this study, which have certain limitations. However, the drug resistance genes detected in this experiment also illustrates the principle of “gene load.” Some studies have shown that the “super-integration antibiotic resistance gene load” may affect the fitness of pathogens, which is consistent with our research conclusions (Guo et al., 2012; Darmency et al., 2015).

## Conclusion

In conclusion, to the best of our knowledge, this study provides the first evidence for polymorphisms within the variable region promoter of class 1 integrons in clinical Proteus isolates. The results indicated that the gene cassette in the integron in Proteus strains confers antibiotic resistance to aminoglycosides, trimethoprim, and chloramphenicol. Class 1 integron-positive Proteus strains generally have strong promoters, and strains with strong promoters are more resistant to amikacin, gentamicin, and tobramycin than strains with weaker promoters, strains with relatively weak integron promoters can “afford” a heavier intra- and extra-integron antibiotic resistance gene load. Importantly, this study also provides the first evidence for the gene cassettes estX and psp in clinical isolates of Proteus. In addition, beta-lactamase genes and PMQR are widely prevalent in clinical isolates of Proteus, mainly blaTEM-1, blaOXA-1 and qnrD and aac (6′)-Ib-cr. Interestingly, it was also found that in Proteus aac(6′)-Ib-cr may be located on transposons, insertion sequences or other mobile genetic elements rather than on integrons, suggesting multiple pathways in its dissemination.

## Author Contributions

LX and QW conceived the study. WL coordinated the study. XW, NK, MC, and LZ performed the experiments. LX and QW analyzed the data and wrote the manuscript. QW and WL revised the manuscript.

## Conflict of Interest Statement

The authors declare that the research was conducted in the absence of any commercial or financial relationships that could be construed as a potential conflict of interest.

## Footnotes

1 http://www.ncbi.nlm.nih.gov/BLAST
